# Endoscopic resection and allografting for benign osteolytic lesions of the calcaneus

**DOI:** 10.1186/s40064-016-2059-y

**Published:** 2016-04-11

**Authors:** Andreas Toepfer, Ulrich Lenze, Ludger Gerdesmeyer, Florian Pohlig, Norbert Harrasser

**Affiliations:** Klinik für Orthopädie und Sportorthopädie, Klinikum rechts der Isar der Technischen Universität München, Ismaningerstr. 22, 81675 Munich, Germany; Department for Orthopaedic Surgery and Traumatology, University Schleswig-Holstein, Campus Kiel, Kiel, Germany

**Keywords:** Foot tumor, Unicameral bone cyst, Bone tumor, Intraosseous lipoma, Minimally-invasive surgery, Ossoscopy, Calcaneal bone cyst, Lipoma of bone, Simple bone cyst, Osteolysis

## Abstract

**Background:**

Both unicameral bone cysts and intra-osseous lipoma of the calcaneus are rare entities which are mostly diagnosed due to unspecific heel pain, pathologic fracture or as an incidental finding. Minimally-invasive ossoscopy with endoscopic resection of the tumor followed by grafting can potentially minimize risks of open surgery and speed up convalescence. The objective of this study is to present a simple, safe and cost-effective surgical technique for endoscopic surgical treatment of benign osteolytic lesions of the calcaneus.

**Description of technique:**

We present our modifications to previously described techniques of endoscopic curettage with a particular focus on intraosseous lipoma. The key point for grafting is the use of a funnel-shaped ear speculum facilitating the plombage with allogenic cancellous bone chips.

**Patients and methods:**

Between June 2013 and January 2015 ten consecutive patients underwent ossoscopy of the calcaneus. There were 4 cases of intraosseous lipoma and 6 cases of unicameral bone cyst. In a retrospective study, radiological results were analyzed using the Glutting-Classification, functional outcome was recorded with the AOFAS Hindfoot score.

**Results:**

Radiographic follow-up and functional outcome showed good to excellent results. All lesions radiologically classified as “healed”. AOFAS score (max. 100 pts) ranged from 74 to 100 (ø94.4 ± 9.3).

**Conclusions:**

This technique is a simple and safe procedure for benign osteolytic bone lesions of the calcaneus. Compared to its alternatives, grafting with allogenic cancellous bone might prove favourable in this localization for several reasons: Osteointegration, handling, availability and costs. Our preliminary investigations show promising results although further clinical and radiographic results are needed.

## Background

**Fig. 1:**
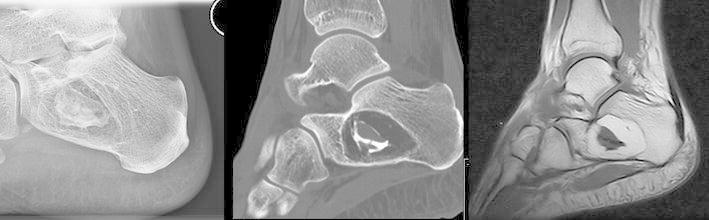
Typical radiographic appearance of calcaneal lipoma of bone: plain radiography (*left*), sagittal CT-scan (*center*) and T1-weighted sagittal MRI (*right*) show an osteolytic lesion with central calcifications surrounded by fat-intense soft-tissue

Intraosseous lipoma and simple bone cyst are the two entities most often responsible for benign osteolytic lesions of the calcaneus. Lipoma of bone is a benign neoplasm of adipocytes that typically arises within the medullary cavity of bone. Intra-osseous lipoma is rare and accounts for less than 0.1 % of primary bone tumors, their actual incidence is not known. Considering that only a few cases of lipoma of bone have been published and often seem to be accidental findings, reliable data regarding age distribution is not available. Based on published reports a mean age of 38 years with a wide age range from 2nd to 8th decade can be determined. Males seem to be affected more frequently than females at a ratio of 4:3. Besides the proximal, metaphyseal femur the calcaneus is a common predilection site (Freyschmidt and Jundt 2010). Intramedullary lipoma may be asymptomatic (30 %) or produce aching pain or swelling (70 %), rarely it presents as a pathologic fracture (Fletcher et al. 2013; Freyschmidt and Jundt 2010). The typical appearance of a calcaneal lipoma consists of a well-defined lytic mass surrounded by a thin rim of sclerosis located in the ventral triangular area between the major trabecular groups (Diard’s Area *6*) (Diard et al. 2007; Weger et al. 2013). The lesion often contains central dystrophic calcifications, commonly described as nidus or sequestrum (Fig. 1). A pathologic fracture of calcaneal lipoma is very rare (Weinfeld et al. 2002).

**Fig. 2:**
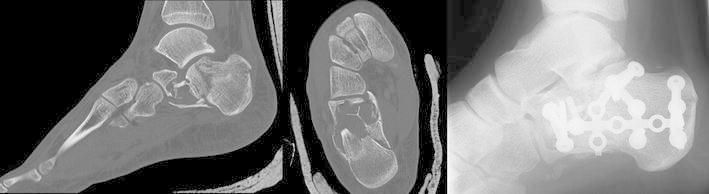
Pathological fracture due to a simple calcaneal bone cyst in a 16-year old male patient. The fracture occurred after jumping down three steps (approx. 40 cm)

Unicameral (simple) bone cyst (UBC) is a common tumor-like lesion of the bone. There is a strong predilection for the long bones of the proximal humerus and proximal femur, accounting for up to 85 % of all cases. The calcaneus is the sixth-most common UBC site, but it is the most common tarsal bone affected (Levy et al. 2015). Localized at the lower extremities, UBC can cause persevering pain and thus justify surgical therapy (Sung et al. 2008). The main indication for treatment is pain and prevention of a pathologic fracture (Fig. 2). Pogoda et al. (2004) proposed that cysts reaching 100 % of the cross-sectional diameter in the coronal plane and 30 % in the sagittal plane were at risk for a pathologic fracture (Yildirim et al. 2011). Regarding localization and presentation on plain radiographs, calcaneal lipoma can be identical to unicameral bone cysts of the heel bone. These findings strictly have to be differentiated from physiological diminution of the calcaneal bone structure in the very same area (De Wilde et al. 2004; Sirry 1951). If a lytic bone lesion of the calcaneus is seen on plain radiographs, MRI is mandatory for further clarification of relevant differential diagnoses (Weger et al. 2013).

Treatment recommendations for *asymptomatic* calcaneal lipoma and unicameral bone cysts are still being discussed controversially. However, surgery is indicated in the presence of pain resistant to conservative treatment methods, impending or pathologic fractures and when a histopathological diagnosis is required for differential diagnoses like aneurysmal bone cyst, giant cell tumor or any other suspected aggressive bone lesion (Ulucay et al. 2009). Depending on clinical and radiographic findings, open biopsy or core needle biopsy versus intralesional tumor resection have to be discussed individually.

The type of intervention for unicameral bone cysts remains controversial (Levy et al. 2015). General treatment options for UBC include curettage in combination with autologous or allogenic grafting, the use of bone substitutes, autologous bone-marrow injection and various methods of cyst decompression including cannulated screws or a cannulated hydroxyapatite pin (Chang et al. 2002; Levy et al. 2015; Pogoda et al. 2004; Shirai et al. 2015; Yildirim et al. 2011). Nowadays most of these techniques can be performed percutaneously or minimally-invasive. For the weight bearing lower extremity, especially for calcaneal localizations, special considerations have to be taken into account, though. In 2015 Levy et al. conducted the first systematic review on the treatment of unicameral bone cysts of the calcaneus. His results clearly demonstrated that all patients who underwent operative curettage with bone substitute—whether autograft or allograft—enjoyed a statistically significant improvement in heel pain and demonstrated the best outcomes in his review regarding osteointegration and rate of recurrence (Levy et al. 2015). Curettage with bone augmentation demonstrated significant improvements over curettage with cannulated-screw placement. No information was available on endoscopic procedures with minimally-invasive curettage and bone grafting. Moreover, curettage with bone graft substitutes showed generally better outcomes than steroid injections (Cho et al. 2007; Glaser et al. 1999; Levy et al. 2015; Mainard and Galois 2006). Standard surgical procedures such as open curettage and autologous bone grafting can entail non-negligible risks and complications, though. Potential donor site morbidity is a major disadvantage of autologous bone grafting (Calori et al. 2014; Schaaf et al. 2010). Conventional open curettage and plombage of a benign lytic calcaneal bone lesion are usually performed through a lateral surgical approach with a longitudinal or L-shaped skin incision and a fenestration of the lateral cortical wall of the calcaneus. Prolonged wound healing and iatrogenic damage of the sural nerve are well-known complications for this localization (Ding et al. 2013; Zhang et al. 2015). Furthermore, a large fenestration can weaken the cortical structures of the calcaneus additionally. Advantages of MIS (minimally-invasive surgery) include less pain, less scarring and better aesthetical results as only a small incision and limited dissection is necessary (Mainard and Galois 2006; Yildirim et al. 2011). Our hypothesis for this procedure is that endoscopic resection and allografting for benign osteolytic lesions of the calcaneus minimizes risks associated with conventional open surgery and autologous grafting whilst offering all the benefits of a minimally-invasive technique and of the traditional treatment with open curettage and grafting in terms of good osteointegration and low rates of recurrence. Endoscopic curettage of solitary calcaneal cysts with different forms of grafting has been reported before with good results (Innami et al. 2011; Yildirim et al. 2011). To the best of our knowledge, endoscopic treatment for intraosseous lipoma (IOL) of the calcaneus has only been reported as a case report so far (Futani et al. 2007; Muramatsu et al. 2014). Compared to autografts allogenic bone is easily available and almost unlimited in supply. Compared to injectable bone substitute, percutaneous grafting with allogenic cancellous bone chips can be exhausting and time-consuming, though. Costs and osteointegration might still favour the use of allografts. We present our modification to previously described techniques of endoscopic curettage with particular focus on IOL and allogenic cancellous bone grafting. The key point of the procedure is the use of a funnel-shaped ear speculum facilitating the filling of the bone cavity with allogenic cancellous bone chips. The objective of this study is to present a simple, safe and cost-effective surgical technique for ossoscopic treatment of benign osteolytic lesions of the calcaneus.

### Surgical technique

**Fig. 3:**
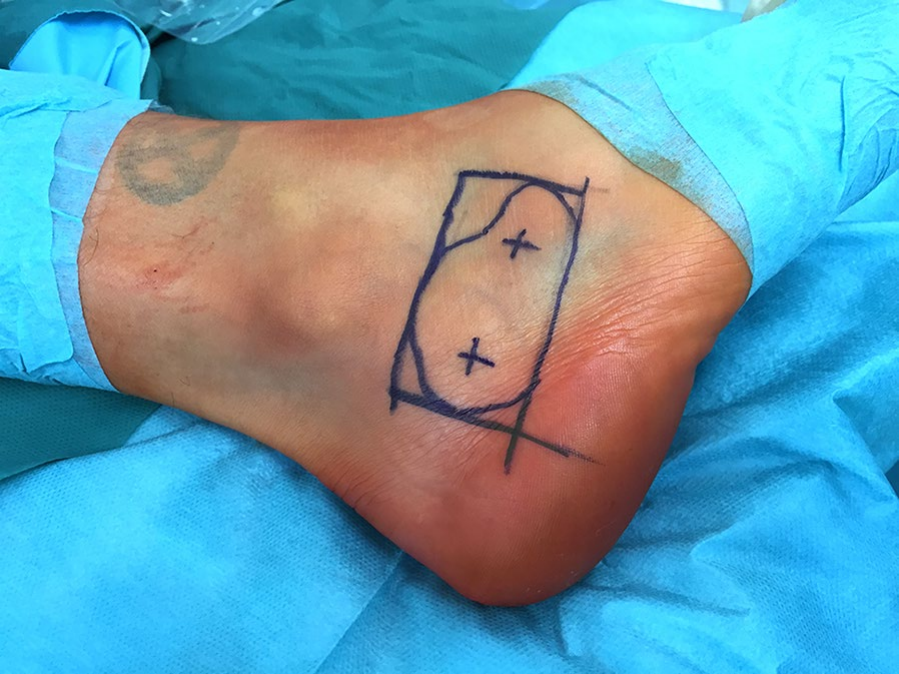
With fluoroscopic control, the margins of the bone lesions and the two portals for ossoscopy are marked on the skin over the lateral calcaneus

**Fig. 4:**
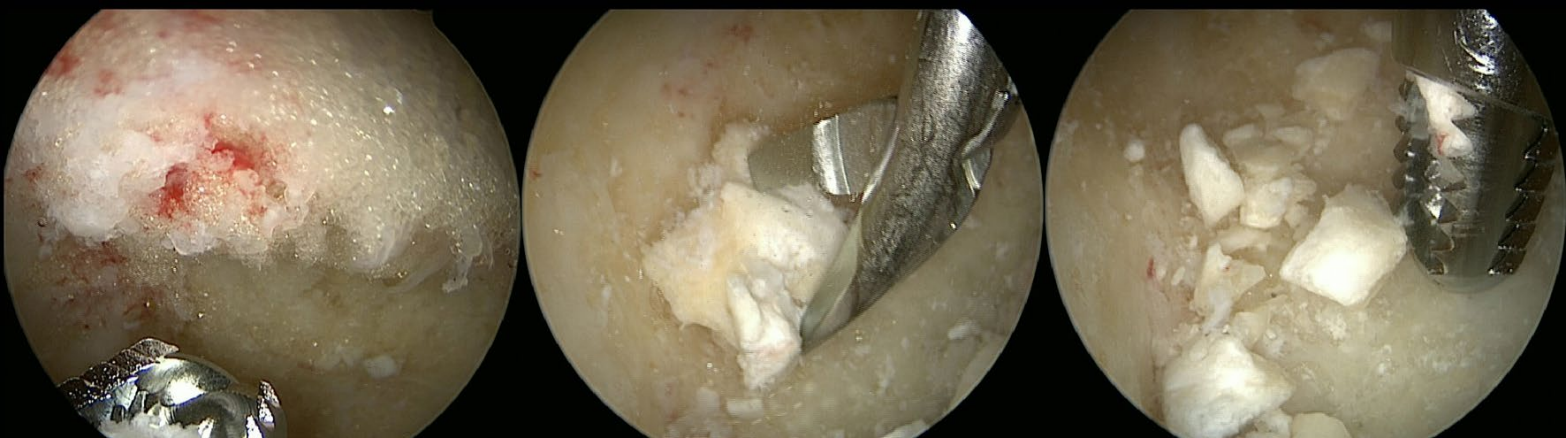
After the introduction of the scope into the bone cavity, vision is often impaired by fat tissue in the case of intra-osseous lipoma. After irrigation and endoscopic removal of the fat tissue with an arthroscopic shaver (*left*), residual calcifications can be identified and removed with an arthroscopic grasper or shaver (*middle* and *right*)

Under general anaesthesia, the patient is put in a stable lateral position on a radiolucent table. The dimensions of the bone lesion are marked under fluoroscopic control on the skin of the lateral rear foot with a sterile pen. Depending on the size of the cystic bone lesion, the two portals for ossoscopy are marked accordingly (Fig. 3). After skin incision and blunt dissection of the underlying soft tissue, the thinned-out cortex can be penetrated with a semi-sharp obturator before the sheath for a 4 mm scope is introduced into the cavity. During blunt dissection to the lateral wall of the calcaneal bone, care must be taken not to harm the sural nerve and the peroneal tendons. Contrary to ossoscopy of the calcaneal bone for unicameral bone cyst, clear vision of the bone cavity can only be achieved after a second portal has been established and thorough endoscopic irrigation is performed. By this, loose lipomatous tissue is washed out and the typical calcified areas of intraosseous lipoma become visible. Calcifications are cleaned out with an arthroscopic shaver, larger pieces can be grasped with an arthroscopic punch or grasper (Fig. 4). Often, a tennisnet-like pseudo-membrane is covering the walls of the cavity. This membrane is common for unicameral bone cyst (UBC) but can also be present in calcaneal lipoma. The membrane is resected and sent for histopathological analysis.

**Fig. 5:**
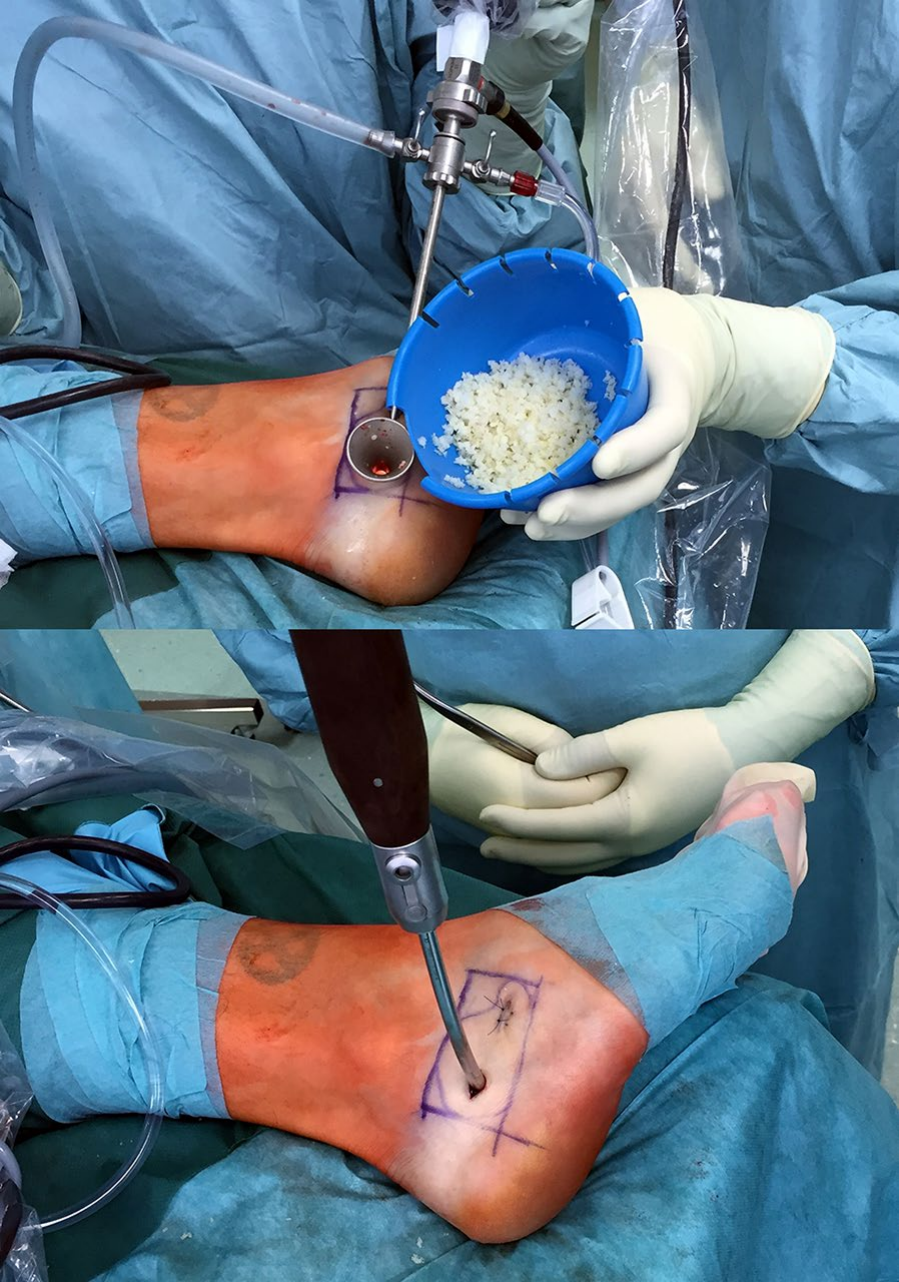
Minimally invasive bone grafting made easy through an ear speculum (*top*). Impaction of the cancellous bone chips with a curved bone tamp (*bottom*)

Before allogenic grafting, the cavity is rinsed with 95 % ethanol as a local adjuvant therapy ensuring denaturation of remaining cyst membranes. The application time for the ethanol should not exceed 2–3 min, and after intermittent thorough irrigation with sterile saline solution, this procedure is repeated 2–3 times. The ethanol should not get in contact with any soft tissue to avoid damage to sensible structures like the sural nerve. For grafting, allogenic cancellous bone is our preferred choice for plombage of the cavity. For easy application of the allogenic cancellous bone chips a small 3 mm *Boucheron* ear speculum (e.g. Aesculap©, Tuttlingen/Germany) is introduced through one of the ossoscopy portals. To our experience, this device has proved to be easier to handle compared to previously used instruments like a pedicle filler (borrowed from spine surgery) or a hollow bone punch, originally used for biopsies. Under endoscopic vision through the second portal, impaction of the bone graft is performed intermittently (Fig. 5). After final fluoroscopic control, both portals can be sealed with a collagen sponge to avoid accidental leakage of the bone graft. After wound closure, the foot is immobilized in a semi-rigid lower leg orthosis.

Partial weight bearing is advised for 6 weeks and impact sports should be avoided for a minimum of 3 months postop. A radiologic follow-up is performed 6 and 12 weeks and 12 months postop with plain radiography. Repeated MRI imaging after complete resection of lipoma of bone is usually not required as recurrence is very rare (Fletcher et al. 2013).

### Patients and methods

Between June 2013 and January 2015 ten consecutive patients with either intra-osseous lipoma (IOL) or unicameral bone cyst (UBC) of the calcaneus were included for ossoscopic, minimally-invasive tumor surgery in a single university tumor institute. Mean follow-up was 19.8 m (min. 9.5; max. 39.3 m). Patient age was 18.57 ± 5.35 years (min. 12.4; max. 28.3 years). There were 7 male and 3 female patients (5 right and 5 left calcanei). In 4 patients intra-osseous lipoma and in 6 cases unicameral bone cyst of the calcaneus was the reason for ossoscopic resection. Seven patients (5 × UBC, 2 × IOL) complained about unspecific heel pain before surgery. Further diagnostics revealed a lytic bone lesion of the heel. Three patients (2 × UBC, 1 × IOL) were indicated for ossoscopy after incidental findings of a bone lesion on plain radiography which was initially performed for ankle sprains. Critical size of the tumor (2 cases) and tumor fear (1 case) were indications for surgery. Patient characteristics can be found in Table 1. Before surgery, all patients had to obtain plain radiography and MRI of the rear foot whereas CT scanning was optional. Eligibility criteria for this procedure were not fulfilled if the size of the osteolysis was less than 3 cm in the sagittal plane as smaller lesions are not suitable for ossoscopy with a standard arthroscope in our experience. All participants were available for clinical and radiological follow-up and completed the functional analysis. Functional outcome was measured using the AOFAS Hindfoot Score. Preoperative scores were not obtained. The radiological result was evaluated using the classification system of Glutting and Chang (Chang et al. 2002). Histological work-up confirmed suspected initial diagnoses in all cases.

**Table 1:** Patient characteristics and operation time

Case number	Age at diagnosis (years)/gender	Presenting circumstances	Side	Entity	Grafting	Operation time (h)
1	14/m	Heel pain	Right	UBC	ACB	1:42
2	12/f	Incidental	Left	UBC	ACB	1:19
3	15/f	Incidental	Left	UBC	ACB	0:47
4	28/m	Heel pain	Left	IOL	ACB	1:06
5	18/m	Incidental	Right	IOL	ACB	1:10
6	16/m	Heel pain	Right	UBC	ACB	1:05
7	34/f	Heel pain	Right	IOL	ACB	1:29
8	15/f	Heel pain	Right	UBC	ACB	0:59
9	15/f	Heel pain	Left	UBC	ACB	0:35
10	26/m	Heel pain	Right	IOL	ACB	1:43

*ACB* allogenic cancellous bone, *IOL* intra-osseous lipoma, *MIS* minimally-invasive surgery, *UBC* unicameral bone cyst

## Results

Mean operation time in our study group was 71.5 ± 22.2 min (min. 35, max. 103 min) (Table 1). A seroma with prolonged superficial wound healing developed in one patient. Repeated blood tests and a smear test of the wound were able to rule out wound infection. No sural nerve irritations or other complications were noticed. All patients were allowed full weight bearing and were able to return to everyday activities without orthopaedic aids 6 weeks after surgery. Radiographic follow-up showed desirable bony consolidation in all cases. There was no persistent cyst and no recurrence. According to the classification of Glutting and Chang, all ten lesions radiologically classified as “healed” with a cyst filled by a formation of new bone with or without small static, radiolucent areas less than 1 cm in size (Chang et al. 2002). Thus, the overall radiological success rate was 100 %. AOFAS score (max. 100 pts) ranged from 74 to 100 (ø94.4 ± 9.3). Within this score, results for pain scored ø 36.6 ± 7.0 (min. 20, max. 40 pts.) with 40pts for no pain and 0pts for constant pain.

## Discussion

At the current time, no other studies involving endoscopic treatment for both IOL and UBC of the calcaneus are available for comparison. In the literature study designs for endoscopic treatment of calcaneal UBC are heterogenous regarding grafting, radiological evaluation and functional testing. Innami et al. (2011) reported similar functional results with an overall AOFAS of ø98.0 ± 4.2 pts and pain score of ø39.0 ± 3.2 for his patients. Grafting was performed with an injectable bone substitute, though. All of their patients returned to their initial levels of sports activities within 8 weeks (Innami et al. 2011).

Mean operation time in Yildirim’s study was significantly shorter than in our study (ø45 min) (Yildirim et al. 2011). No information on intraoperative local adjuvant therapy (e.g. 95 % ethanol) was found in his work. The operation time in our patients diminished significantly over the course of time with more experience in the ossoscopic procedure and after the introduction of the speculum. We experienced that meticulous ossoscopic curettage of IOL with resection of dystrophic calcifications was more time-consuming than in UBC. Operation time of our procedure might be additionally cut down by the use of injectable bone substitute but, in our experience, osteointegration is inferior to allogenic bone, adverse effects have been reported (Lee et al. 2002) and, last but not least, cost-effectiveness is mostly disadvantageous (Kurien et al. 2013). Compared to autologous bone or bone substitute, allogenic bone is easily available, affordable and can offer good osteoinductive and osteoconductive properties (Oryan et al. 2014). The major advantage is to avoid sacrificing host tissue and the challenges of donor site morbidity (Greenwald et al. 2001). Disadvantages of allogenic bone grafting include that harvesting and conservation of allogenic grafts might be a limiting factor in some countries (Nandi et al. 2010; Pruss 2007). Moreover, there are two theoretical concerns regarding the use of bone allografts: Antigenicity and the risk of disease transmission. With modern means of tissue processing these risks seem to be almost negligible (Grover et al. 2011; Nasr et al. 2000).

The systematic review conducted on treatment options for calcaneal bone cysts by Levy et al. found no clear distinction between autografting and allografting regarding a difference in pre- and post-operative heel pain. Although cysts treated with autografts consolidated  on radiography at a significantly greater rate than those treated with allografts, no recurrences, complications or reactions suggestive of graft rejection were recorded in either group (Levy et al. 2015).

Converted to USD the price for the allogenic cancellous bone that is applied in our patients is 323$ USD for 10 ml (DIZG©, Germany). Alternatively, injectable bone substitutes that were used in our clinic for similar indications previously included different combinations of calcium sulphate, calcium phosphate and hydroxyapatite (Cerament/Bonesupport©, 644$ USD for 10 ml; Pro-Dense/Wright Medical©, 497$ USD for 10 ml; Arthrex/QuickSet©, 456$ USD for 5 ml or 520$ USD for 8 ml).

In our experience, the use of calcium phosphate cement, as proposed by other authors (Innami et al. 2011), is not advisable for permanent use in all localizations. This applies all the more for young patients as encountered in these entities. Bone cement, either (potentially) bioresorbable or not, might eventually thin-out the surrounding cortices in a non-cancellous environment. This phenomenon is well-known in other areas where bone cement is being used for temporary filling after tumor resection (e.g. distal radius in cases of giant cell tumor) (Gaston et al. 2011). Long-term results will need to prove its safety regarding osteointegration and potential diminution of bone stock. Osseous integration of the bone graft seems not to be sped-up by an ossoscopic procedure compared to traditional open procedures (Yildirim et al. 2011). In either case, partial weight bearing is advised for a minimum of 6 weeks postop. In contrast, the instant strength of most injectable bone substitutes could allow for full weight bearing immediately after the surgery.

After endoscopic resection of IOL histopathological confirmation of the diagnosis can prove to be difficult. Continous irrigation is needed to obtain a clear vision of the cavity and all lipomatous tissue will be washed out promptly. Where present, dystrophic calcifications can be extracted and sent for histopathological analysis. Alternatively, a specimen of lipomatous intra-osseous tissue can be saved for further analysis once the first portal for ossoscopy has been established. Without endoscopic vision and irrigation, a percutaneous biopsy can produce enough representative tissue for a precise histopathological diagnosis in most cases. For UBC, the inner lining of the bone cavity will not be washed out by endoscopic irrigation and samples can easily be saved for further diagnostics by ossoscopic resection.

We encountered one postoperative complication with a seroma and delayed wound healing in our follow-up, accounting for a 10 % complications rate (1/10). With the same number of patients included in his follow-up (n = 10), Innami et al. (2011) did not find any complications related to the surgery consisting of endoscopic resection and calcium phosphate cement plombage for calcaneal UBC. The same applies to a study performed by Yildirim et al. (2011) comparing open versus endoscopic curettage and grafting. Here, no complications were recorded in the group with percutaneous endoscopic curettage. Compared to these studies the 10 % complication rate in our series seems relatively high and might be explained by the small sample size of both Innami’s (10 patients), Yildirim’s (13 patients) and our own study group (10 patients). For open procedures, some authors report higher complications rates (Yildirim et al. 15.4 %, Yildirim et al. 2011), others no complications whatsoever (Park et al. 2008; Polat et al. 2009).

Limitations of this study include the small sample number, the short follow-up (ø19.8 m) and the retrospective study design without control cases. A randomized controlled study with a larger sample number will be necessary for the future comparing injectable bone substitute and allogenic cancellous bone grafting for ossoscopic treatment of calcaneal UBC and IOL. The nature of calcaneal UBC and IOL, often unsymptomatic and rare, will hinder this project, though.

We recommend that small, symptomatic benign osteolytic lesions of the calcaneus with a diameter of 3 cm or less in the sagittal plane are better suited for mini-open surgery as standard instruments for ossoscopy will interfere with each other and complicate handling. Moreover, both portals can brake out and form one large opening due to their close proximity to each other. Alternatively, the use of a 2.7 mm scope is a viable option for very small osteolytic bone lesions.

## Conclusion

Musculoskeletal tumors and tumor-like lesions of the calcaneus are rare but have to be taken into consideration for chronic heel pain. Once a lytic bone lesion is recognized on plain radiography, calcaneal lipoma of bone and unicameral bone cyst represent one of several entities that must be further investigated by MRI (Pogoda et al. 2004). To avoid pathologic fractures, large lesions are recommended for prophylactic surgical therapy, even if the patient is free of symptoms (Pogoda et al. 2004). Traditionally, symptomatic cases of IOL or UBC were treated with open curettage and bone grafting. Compared to open curettage with a longitudinal incision parallel to the planta pedis or a classic L-shaped incision, minimally-invasive ossoscopy can diminish the risk of impaired wound healing and speed-up superficial wound healing (Yildirim et al. 2011). Several studies demonstrated that minimally-invasive ossoscopy can offer a safe alternative to open surgical procedures (Futani et al. 2007; Innami et al. 2011; Mainard and Galois 2006; Yildirim et al. 2011). Compared to endoscopic resection and filling with an injectable bone substitute, allogenic bone grafting is both less expensive (Kurien et al. 2013) and might prove biologically superior. As a modification of previously reported procedures, the use of an ear speculum, as described in our technique, can facilitate the process of grafting with cancellous bone chips.
